# The Impact of Macronutrient Ordering on Postprandial Glycaemic Control in Diabetes: A Systematic Review

**DOI:** 10.1002/edm2.70228

**Published:** 2026-04-27

**Authors:** Rebecca McKenzie, Charlotte Sterling, Hannah O'Hara, Jayne Woodside

**Affiliations:** ^1^ Centre for Public Health, School of Medicine, Dentistry and Biomedical Science Queen's University Belfast Belfast Northern Ireland UK

**Keywords:** diabetes, dietary pattern, glycaemic control

## Abstract

**Aims:**

This systematic review aims to investigate if macronutrient ordering is effective at lowering postprandial glucose excursions in individuals with diabetes.

**Methods:**

A systematic search of Medline, Embase and Web of Science was conducted up to February 2025. Two independent reviewers screened title and abstract using Covidence software. Data extraction and risk of bias assessment were performed by one reviewer and checked by a second reviewer. Findings were synthesized narratively. PROSPERO registration: CRD42025639742.

**Results:**

In total, we included six studies involving 144 participants. Most studies reported that a carbohydrate last meal pattern was effective at lowering postprandial glucose excursions in individuals with diabetes. There was some evidence for the carbohydrate last meal pattern lowering insulin levels and increasing GLP‐1 and GIP levels in individuals with diabetes. The risk of bias ranged from low to some concerns, and the GRADE assessment showed a low certainty of evidence.

**Conclusions:**

Evidence suggests that the carbohydrate last meal pattern effectively reduces postprandial glucose excursions in individuals with diabetes. However, significant heterogeneity in study design limits overall certainty in the findings. Further research is needed to confirm these effects.

## Graphical abstract

Consuming carbohydrates last, after protein and vegetables, is associated with reduced postprandial glucose excursions in individuals with diabetes. This systematic review of six studies suggests macronutrient ordering may be a simple, effective strategy to improve glycaemic control.

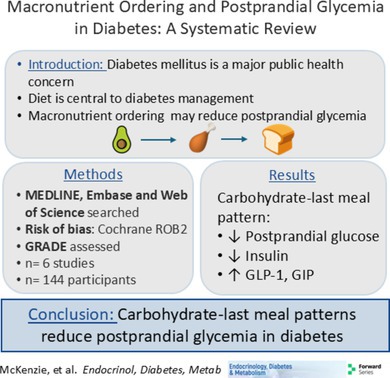

## 1 Introduction

Diabetes Mellitus (DM) is a major public health concern, with 4.7 million people in the UK living with the condition [1]. Diabetes UK predicts that, by 2030, this number will exceed 5.5 million, as the incidence of diabetes continues to rise due to an ageing population and increasing rates of obesity [2]. The NHS spends around £10 billion a year on diabetes, which is equivalent to 10% of its budget [1]. In the UK, 90% of people living with diabetes have type 2 (T2DM), and 7% have type 1 diabetes (T1DM) [1]. Furthermore, 1 in 20 pregnancies are affected by gestational diabetes mellitus (GDM), and these women are four times more likely to develop T2DM in their lifetime [3, 4]. If not adequately managed, all forms of diabetes can lead to complications such as cardiovascular disease, nephropathy, neuropathy, and retinopathy, and is a major contributor to mortality [5].

Diet plays a key role in the management of diabetes, particularly in regulating postprandial glucose (PPG). Humans spend approximately 16 h per day in the postprandial state, making it important in managing glucose levels in people with diabetes [6]. PPG is an important contributor to hyperglycaemia, which is a risk factor for developing many of the adverse outcomes associated with diabetes [7]. Postprandial hyperglycaemia is 38% ± 4% more prevalent in individuals with T2DM compared to glucose‐tolerant individuals [6].

Macronutrient ordering—consuming meal components in a specific sequence—has recently gained attention as a strategy to lower PPG levels. Several mechanisms have been proposed to explain this, including reducing the rate of gastric emptying, and secretion of insulin and incretin hormones such as glucagon like peptide‐1 (GLP‐1), which may attenuate sharp postprandial glucose excursions [6]. Recently, studies have been carried out in both healthy individuals and people with diabetes that show promising effects of ordered eating on postprandial hyperglycaemia [7]. Furthermore, it has also been demonstrated that following a carbohydrate‐last meal consumption pattern is beneficial in the longer term for glycaemic control in patients with diabetes [8]. Macronutrient ordering potentially offers a simple, effective, safe and inexpensive way to help improve blood glucose control in people with diabetes and reduce associated complications. However, despite growing interest in this area, no systematic review to date has focussed exclusively on individuals with diabetes. Existing reviews have either included healthy or mixed populations [7, 9]. These reviews concluded that consuming protein and/or vegetables before carbohydrates acutely reduces postprandial glucose and insulin excursions, though most evidence is derived from short‐term studies. Furthermore, other studies which evaluated response in participants with diabetes have used a method involving ‘nutrient preloading’, where non‐carbohydrate macronutrients (namely protein and fat) are consumed separately prior to a meal rather than as part of the meal sequence itself [10]. Although nutrient preloading has been shown to reduce postprandial hyperglycaemia in both T2DM and at‐risk individuals, it represents a conceptually different dietary strategy than macronutrient ordering. Macronutrient preloading involves additional caloric intake, therefore may contribute to body weight gain [10]. This review therefore focuses specifically on macronutrient ordering within meals, rather than the addition of separate premeal supplements or food.

The primary objective of this systematic review was to examine the effectiveness of macronutrient ordering as an intervention to lower postprandial glucose levels in individuals with diabetes. The secondary objective was to examine the effect of macronutrient ordering on blood insulin levels.

## 2 Methods

### 2.1 Registration

The review protocol was prospectively registered with PROSPERO: International Prospective Register of Systematic Reviews in February 2025 (CRD42025639742).

### 2.2 Search Strategy

Electronic literature searches were conducted using MEDLINE, Embase, and Web of Science. The search strategy used combinations of the following terms: *macronutrient*, *food*, or *meal*; *order* or *sequence*; *postprandial glycaemia*, *glycaemic response*, or *glucose excursion*; and *diabetes*. Boolean operators AND/OR were used to combine these terms. The search was conducted in February 2025 and there were no search restrictions. The full database search strategies are provided in Appendix S1. In addition to the initial database searches conducted in February 2025, a citation alert was established in Web of Science using the same search strategy to identify newly published studies during the review process. Records identified through this alert were screened using the same eligibility criteria as the original search. One additional study identified through this process met the inclusion criteria and was included in the final synthesis. Grey literature sources (e.g., conference proceedings, dissertations and trial registries) were not systematically searched, as the review aimed to include peer‐reviewed full‐text studies to ensure sufficient methodological and outcome reporting for critical appraisal and synthesis.

### 2.3 Eligibility Criteria

#### 2.3.1 Inclusion Criteria

Adults ≥ 18 years diagnosed with type 1, type 2, or gestational diabetes based on recognized diagnostic criteria; Studies examining macronutrient ordering on glycaemic control; Studies that compare intervention to another intervention or control meal; and randomized controlled trials (RCTs).

#### 2.3.2 Exclusion Criteria

Studies focusing on healthy individuals without diabetes or with prediabetes; studies that do not investigate macronutrient ordering; studies with no comparison or control; studies not reporting postprandial glucose; studies investigating isolated macronutrient preloads administered prior to a meal; and observational studies, case reports, editorials, narrative reviews, or animal studies.

Studies investigating isolated macronutrient preloads administered prior to a meal were excluded, as these represent a distinct dietary intervention from macronutrient ordering within a meal.

### 2.4 Study Selection

The search results generated from each database were imported into the Covidence online software platform (Covidence—Better systematic review management) to systematically sort the papers. Two independent reviewers (RM and CS) screened the titles and abstracts and full texts to establish which studies were suitable for inclusion. Any disagreements were resolved by a third independent reviewer (JW). Reference lists of included studies were also reviewed for additional relevant citations.

### 2.5 Data Extraction

Data was extracted by one reviewer (RM) using Covidence and checked by a second reviewer (CS). Any disagreements were resolved by a third independent reviewer (JW). A standardized form was used to extract the following information from each study: study design, population characteristics, intervention and comparator details, outcome measures (postprandial glucose, insulin levels), and risk of bias assessments. Where studies included mixed populations (e.g., healthy individuals and participants with type 2 diabetes), only the data relating to participants with type 2 diabetes were extracted and analysed. For the purposes of this review, macronutrient ordering was defined as the sequential consumption of different macronutrient components within a single meal.

### 2.6 Risk of Bias Assessment

The risk of bias was assessed by the principal author (RM) using the Cochrane Risk of Bias 2 tool (ROB2) [11]. RoB 2 is a validated, structured tool developed by Cochrane to evaluate the internal validity of randomized controlled trials. The domains assessed by this tool include bias arising from the randomization process; bias due to deviations from intended interventions; bias due to missing outcome data; bias in measurement of the outcome; and bias in selection of the reported result. The judgement options include high risk of bias, some concerns and low risk of bias. Judgement is determined using the signalling questions provided in the Cochrane Handbook. Covidence software was used to implement the ROB2 tool.

### 2.7 Evidence Quality Assessment

The certainty of evidence for each outcome was assessed using the GRADE (Grading of Recommendations Assessment, Development and Evaluation) approach, considering risk of bias, inconsistency, indirectness, imprecision and publication bias [12]. The results of the GRADE assessment are presented in Table S1.

### 2.8 Data Synthesis

The outcomes were not suitable for meta‐analysis due to the heterogeneous nature of the study designs and data presentation; therefore, a narrative synthesis was used to appraise these studies.

## 3 Results

### 3.1 Study Selection

**FIGURE 1:**
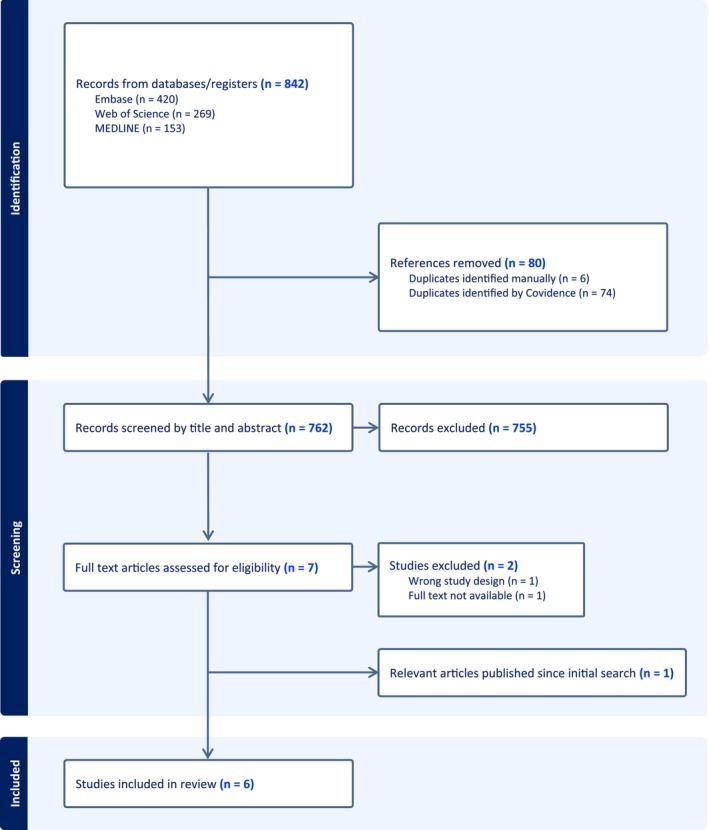
PRISMA flow diagram of the study selection procedure.

The initial database search retrieved 842 potential studies for inclusion. Following the removal of duplicate studies, 762 studies were identified and screened for title and abstract. Of these, seven full texts were assessed and five were eligible for inclusion. One study was excluded because the full text was not available. This study was published only as a conference abstract, and two attempts were made to contact the authors to obtain the full manuscript; however, no response was received. One additional paper was included that was published after the search was conducted. Full details of the process are presented in the PRISMA flowchart, including reasons for exclusions (Figure 1).

### 3.2 Study Characteristics

An overview of each included study is presented in Table 1. Of the six included studies, four were randomized crossover trials, and two were parallel, randomized controlled trials. Two of the studies were conducted in Japan, one in the USA, one in Italy, one in China and one in India. The total sample size was 144 participants, of which 44 were male and 100 female, with individual studies ranging in size from 12 to 54 participants. Participant age varied from 26 to > 65 years old. Of the six studies, four investigated participants with T2DM and two investigated participants with GDM. The length of the interventions varied from 3 days to ~43 weeks, and the meal sequences investigated included carbohydrate first, carbohydrate last and mixed meal patterns. Three of the studies had different groups, two with a control group and an experimental group [15, 17], and one with six different groups for all of the meal patterns investigated [18]. The studies included were published between 2013 and 2025. The retention rate of three of the studies was 100% [13, 14, 16], one study was 87.1% [15], one study was 85% [17], and one study was 83.3% [18].

**TABLE 1:** Overview of intervention studies which examined macronutrient ordering in diabetes.

Authors	Study location	Study design	Meal sequence	Groups	Diabetes status	Sample size	Sex	Mean age ± SD (years)	Withdrawals	Trial duration	Limitations
Imai et al. (2013) [13]	Japan	Randomized controlled crossover trial	Carbohydrate first Carbohydrate last	N/A	Type 2 diabetes	19 (crossover)	6 male, 13 female	65.5 ± 9.4	0	3 days	Small sample size
Kuwata et al. (2016) [14]	Japan	Randomized controlled crossover trial	Carbohydrate first Meat first carbohydrate last Fish first carbohydrate last	N/A	Type 2 diabetes	22 (10 healthy volunteers, 12 patients with Type 2 diabetes (crossover))	Controls: 10 males Type 2 diabetes: 9 male, 3 female	Type 2 diabetes: 59.7 ± 9.7 Controls: 38.4 ± 4.9	0	3 days	No rice before meat arm
Murugesan et al. (2025) [15]	India	Parallel, randomized controlled trial	Fibre first, then protein then carbohydrates Regular diet	Control diet Experimental diet	Gestational diabetes	62 (31 control group, 31 experimental group)	Female	Control‐ 27.26 ± 2.78 Experimental‐ 26.93 ± 2.51	Control group‐ 4 due to lack of response and discontinuing intervention Experimental group‐ 4 due to lack of response and discontinuing intervention	From 24–28 weeks gestation to 4 weeks postpartum (~43 weeks)	Small sample size An open label design with no randomization or blinding Dietary adherence was self‐reported which might be subject to recall bias, underreporting, or misreporting The short‐term nature of follow up Other confounding factors not monitored or adjusted for
Shukla et al. (2017) [16]	USA	Randomized controlled crossover trial	Carbohydrate first Carbohydrate last Mixed meal	N/A	Type 2 diabetes	16 (crossover)	7 male, 9 female	57.7 ± 7.6	0	3 days	Small sample size Unclear generalizability to meals with different macronutrient compositions and patient populations
Tricò et al. (2016) [17]	Italy	Parallel, randomized controlled trial	Carbohydrate last (after high protein, high fat foods)	1. Control diet 2. Experimental diet	Type 2 diabetes	17 (9 control group, 8 experimental group)	Control group‐ 6 males and 3 females, Experimental diet‐ 6 male and 2 females	Control‐ 64 ± 8, Experimental Group‐ 65 ± 7	3 due to poor compliance with study protocol	12 weeks	High variability due to real‐life setting and small population size
Yong et al. (2022) [18]	China	Randomized controlled crossover trial	Carbohydrate first Carbohydrate last Carbohydrate in the middle Three meals a day ad libitum Six meals a day ad libitum	(A) dish‐ > carbohydrate‐ > soup (B) Carbohydrate‐ > dish‐ > soup. (C) soup first, followed by dish and carbohydrate last. (D) Three meals a day ad libitum. (E) Six meals a day ad libitum	Gestational diabetes	12 (crossover)	Female	30.10 ± 3.19	2 due to hyperglycaemia and having 1 remaining test meal	5 days	Small sample size No wash out day No mechanism study

### 3.3 Intervention Characteristics

A description of each intervention along with a summary of the key findings is presented in Table 2. The number of meals consumed for each meal pattern ranged from one to three meals per day and the timing between the consumption of the individual meal components ranged from 0 to 15 min. The total number of calories consumed throughout the intervention ranged from 1924 kJ to 7531.2 kJ. For the measurement of glucose, two of the studies used a continuous glucose monitoring system [8, 18] while four studies used blood samples and a range of assays [14, 15, 16, 17]. Four of the studies reported glucose results in mmol/L and two used mg/dL. The timing of the glucose measurements varied across the studies, one of the studies used 15 min intervals from 0 to 240 min [14], two of the studies used 60 min intervals from 0 to 120 min [13, 15], one study used 30 min intervals from 0 to 180 min [16], one study used 15 min intervals from 0 to 180 min [18] and one study took one glucose measurement at the end of every meal [17]. Four out of the seven studies measured participants adherence to the meals, one of the studies used the mobile application JotForm (a mobile health application which tracks dietary recall and monitors participants adherence to the food ordering regimen) [15], two of the studies had a researcher closely monitor the participant to ensure that they finished the meal in its entirety within the allotted time [16, 18] and one study had participants fill in an ad hoc designed form at each meal [17].

**TABLE 2:** Characteristics of the interventions delivered.

Authors	Description of intervention	Length of Intervention	Number of meals for each meal pattern	Meal components	Timing between individual meal components (minutes)	Total calories (kJ)	Meal duration	Glucose measurement timepoints	How glucose was measured	Glucose measurement units	Other outcomes of interest	Other glucose outcomes reported	Summary of key findings
Imai et al. (2013) [13]	Participants consumed test meals (breakfast, lunch and dinner) of vegetables before carbohydrates and carbohydrates before vegetables for all meals on the 2nd and 3rd day of the study in a randomized crossover design	3 days	1	Vegetables, meat or fish and rice or bread	10	differed between participants (125.6 kJ kg^−1^ per day)	Not stated	60 and 120 min	Continuous glucose monitoring system (Medtronic minimed gold)	mmol/L	None	Mean plasma glucose, mean amplitude of glucose excursions, largest amplitude of glucose excursions, iAUC0‐3 h of each meal, incremental glucose peaks of each meal and mean incremental glucose peak	Eating vegetables before carbohydrates‐> ↓ in standard deviation (*p* < 0.01), ↓ mean amplitude of glycaemic excursions (*p* < 0.01), ↓ largest amplitude of glycaemic excursions (*p* < 0.01) ↓ 1‐h postprandial plasma glucose of breakfast (< 0.05) ↓ iAUC0‐3 h of lunch and dinner (*p* < 0.05) ↓mean iAUC0‐3 h (*p* < 0.05) ↓Incremental glucose peak (*p* < 0.001).
Kuwata et al. (2016) [14]	Participants subjected to meal sequence tests in the morning after an overnight fast on 3 separate days under 3 separate conditions: Fish before rice, rice before fish, and meat before rice	3 days	1	boiled mackerel or grilled beef and steamed rice	15	1924	Not stated	0, 15, 30, 60, 90, 120, 240 min	Blood samples drawn and assessed using standard assays	mmol/L	insulin, c‐peptide, glucagon, and gastric emptying rate	iAUC(−15‐240 min)	Carbohydrate last meal patterns‐ supressed rapid glucose elevation from 30–90 min ↓iAUC compared to CF pattern (type 2 diabetes, FR 1.94 ± 0.22 mmol/L, MR 1.68 ± 0.18 mmol/L, RF 2.77 ± 0.24 mmol/l; healthy, FR 0.95 ± 0.21 mmol/L, MR 0.83 ± 0.16 mmol/L, RF 1.18 ± 0.27 mmol/L) ↓Insulin and C‐peptide levels ↑GIP and GLP‐1 Delayed gastric emptying rate.
Murugesan et al. (2025) [15]	Participants allocated into two groups: control or experimental. Experimental group: structured dietary plan with a specific food sequence: fibre first, followed by proteins and fats, carbohydrates last. Adherence monitored via a mobile health application. Participants followed the diet until delivery. At 4 weeks postpartum, questionnaire administered to determine the long‐term effects of the intervention. The control group was offered general advice on healthy eating but with no specific dietary order. They were also not required to use mobile tracking tools. The postpartum questionnaire was administered to the control group.	From 24–28 weeks gestation to 4 weeks postpartum (~43 weeks)	3 meals a day with 3 snacks until delivery	Vegetarian‐ snacks included nuts, fruit, and vegetables. Typical breakfast included fruit, vegetables, wheat or millet. Lunch included vegetables, sambar or curd, and rice or millet. A typical dinner included vegetable soup, salad, curry or dhal with millet, wheat or chapati. Non‐vegetarian‐ Snacks included nuts, fruit and vegetables. A typical breakfast included fruits and vegetables, omelette or dhal with wheat or millet. Lunch could be sambar, fish or chicken curry, steamed fish, and boiled egg whites with rice or millet. A typical dinner included the same foods as lunch with the addition of dhal.	None	Vegetarian‐ ~1118–1746.5 kcal. Non vegetarian‐ ~1241–1973.5 kcal.	Not stated	0, 60 and 120 min	Glucose oxidase enzymatic method using Beckman Coulter DxC 700 AU analyser	Mg/dL	None	None	Experimental group: Improvements in 1 h PPBG↓ 8.41 mg/dL (*p* < 0.001) and 2 h PPBG ↓ 7.56 mg/dL (*p* < 0.001) FBG showed no significant changes
Shukla et al. (2017) [16]	All participants consumed isocaloric meals of the same composition on 3 separate days under 3 separate conditions: Carbohydrate first, carbohydrate last and all meal components together as a sandwich	3 days	1	Orange juice, ciabatta bread, butter, chicken breast, lettuce, tomatoes, cucumber, and salad dressing	10	2403.42	30 min	30 min intervals up to 180 min	Blood samples drawn from an in‐dwelling venous cannula and glucose concentrations assessed in whole blood using a quantitative enzyme photometry cassette	mg/dL	Insulin and GLP‐1	iAUC(0–180 min)	Postprandial mean glucose concentrations significantly ↓ (20.8%, 30.2%, and 23.1% at 30, 60 and 90 min respectively) following carbohydrate last pattern. iAUC 53.4% lower (*p* < 0.001) following the carbohydrate last meal order compared to the carbohydrate first meal order. Carbohydrate last meal order‐> ↓ postprandial glucose levels compared with the sandwich meal order. Carbohydrate last meal order‐>lower insulin excursions (*p* = 0.003) and increased GLP‐1 response (*p* = 0.19)
Tricò et al. (2016) [17]	Participants randomized to 2 different groups and asked to follow an 8‐week diet after 4‐week run‐in: Control diet‐ standard balanced mild hypocaloric dietary plan with the food composition of 3 typical meals and a table of substitutions with variable equicaloric amounts of different foods. Experimental diet‐ same diet plan in terms of quantity and quality and in addition indications on macronutrient composition of foods and strongly recommended to fix the sequence of macronutrient ingestion at each main meal to eat high‐carbohydrate containing foods last	12 weeks	3 per day for 8 weeks	typical main meal was meat, vegetables, bread, pasta, or fruit	Not mentioned	N/A	Not stated	one measurement after breakfast, lunch, and dinner Measurement Timepoints: 4 weeks before, at baseline, 4 weeks after and 8 weeks after the diet began	Blood samples drawn and assessed using standard assays and by glucometer once a week	mmol/L	None	Fasting plasma glucose, mean glucose	After 8 weeks, experimental diet‐> improved overall glucose control (reduced glycated haemoglobin). 1.0 mmol/L decrease in fasting plasma glucose and 0.8 mmol/L in mean lunch and dinner glucose marked reduction of postprandial glucose excursions (lunch: −1.8 mmol/L *p* < 0.01; Dinner: ‐ 1.0 mmol/L *p* < 0.04).
Yong et al. (2022) [18]	All participants consumed isocaloric meals for 5 consecutive days at breakfast, lunch and dinner. Meal sequences divided into 5 groups: (A) dish first, followed by carbohydrate, then soup last. (B) Carbohydrate first, followed by dish and soup last. (C) soup first, followed by dish and carbohydrate last. (D) 3 meals a day ad libitum. (E) 6 meals a day ad libitum	5 days	3	Breakfast‐ steamed bread, skim milk, boiled eggs. Lunch‐ black rice, seaweed soup, shredded chicken, green bean sprout, Chinese flowering cabbage, oil, beef fillet, dressing. Dinner‐ black rice, seaweed soup, firm tofu, miniature cabbage, olive oil, shrimp meat, dressing. Extra meals‐ steamed bread and 2 meals of boiled corn	None	7531.2	1 h	15‐min intervals from 0 to 180 min after each meal	Continuous glucose monitoring system (Medtronic minimed gold)	mmol/L	None	Fasting plasma glucose, peak glucose and time to peak for each meal, largest glucose level, smallest glucose level, largest and mean amplitude of glycaemic excursions and iAUC0‐3 h for each meal.	Significantly higher peak glucose levels after breakfast and lunch in the carbohydrate first group vs. the carbohydrate last group (*p* < 0.05). Mean glucose level, the largest glucose level, and the area under the curve of the 3‐h postprandial were significantly ↓ in CL groups compared to CF group (*p* < 0.05) Increasing the number of meals ↓ the standard deviation, mean amplitude of glycaemic excursions, the largest amplitude of glycaemic excursions, coefficient variant, and peak glucose level (*p* < 0.05).

### 3.4 Risk of Bias Within Studies

A summary of the risk of bias assessment for each study is presented in Table 3. The quality of each study ranged from some concerns to low risk of bias.

**TABLE 3:** Risk of bias assessment for included studies using Cochrane Risk of Bias Tool 2.

Study	Randomization process	Deviations from intended interventions	Missing outcome data	Measurement of the outcome	Selection of overall results	Overall risk of bias
Imai et al. (2013) [13]	Some concerns	Some concerns	Low risk	Some concerns	Low risk	Some concerns
Kuwata et al. (2016) [14]	Some concerns	Some concerns	Low risk	Low risk	Some concerns	Some concerns
Murugesan et al. (2025) [15]	High risk	Some concerns	Low risk	Low risk	Low risk	Some concerns
Shukla et al. (2017) [16]	Low risk	Low risk	Low risk	Low risk	Low risk	Low risk
Tricò et al. (2016) [17]	Some concerns	Some concerns	Low risk	Low risk	Some concerns	Some concerns
Yong et al. (2022) [18]	Some concerns	Low risk	Low risk	Low risk	Some concerns	Low risk

### 3.5 Synthesis of Results

#### 3.5.1 Glucose Outcomes

A summary of glucose related outcomes is presented in Table 4. Six studies assessed changes in postprandial glucose levels, four of which measured mean plasma glucose (MPG). Of these, three out of four studies (75%) reported a significant reduction in MPG following a carbohydrate‐last meal pattern [13, 16, 17, 18]. In the study by Tricò et al., the carbohydrate last meal pattern produced a 0.8 mmol/L (95% CI (−1.4/−0.2)) reduction in the mean plasma glucose. Similarly, in the study by Yong et al., the carbohydrate last meal pattern produced a reduction in MPG of 0.67 mmol/L compared to the carbohydrate first meal pattern, and in the study by Shukla et al., the MPG was significantly decreased by 20.8%, 30.2%, and 23.1% at 30, 60, and 90 min. In contrast, the study by Imai et al. found a reduction in MPG that did not reach statistical significance [13]. A reduction of 0.15 mmol/L was recorded by Imai et al. Two of the studies that reported a significant reduction in MPG and the study which did not demonstrate a statistically significant reduction used a randomized crossover design [13, 16, 18]. In contrast, the other study used two parallel groups (experimental and control) [17]. There was also heterogeneity in comparator arms. While all studies included a carbohydrate‐last arm, only three directly compared carbohydrate‐last with carbohydrate‐first [12, 14, 15]. This design offers the clearest mechanistic insight, since the only variable altered is meal sequence. In contrast, the parallel group studies compared carbohydrate‐last with a regular diet, which limits mechanistic conclusions but may better reflect clinical practice [15, 17].

**TABLE 4:** The effect of macronutrient ordering interventions on each glucose outcome reported and whether it was statistically significant in participants with type 2 diabetes or gestational diabetes mellitus.

Outcome	Imai et al. (2013) [13]	Kuwata et al. (2016) [14]	Murugesan et al. (2025) [15]	Shukla et al. (2017) [16]	Tricò et al. (2016) [17]	Yong et al. (2022) [18]
Mean plasma glucose	NS	NM	NM	↓*	↓*	↓*
Amplitude of glucose excursions	↓**	NM	NM	↓***	↓**	↓*
1 h PPG	↓*	NM	↓***	↓*	NM	NM
iAUC	↓***	↓*	NM	↓***	NM	↓*
Incremental glucose peaks	↓***	↓*	NM	↓***	NM	NM

*Note:* Statistical significance, ***p < 0.001, **p < 0.01, *p < 0.05.

Abbreviations: NM, Not Measured; NS, not significant.

Three studies evaluated the amplitude of glucose excursions, all studies (100%) demonstrating significant decreases under the carbohydrate‐last condition [13, 16, 18]. Amplitude of glucose excursions reflects the magnitude of glucose fluctuations during the postprandial period, typically defined as the difference between peak and lowest glucose values. This differs from incremental area under the curve (iAUC), which represents the total postprandial glucose exposure over time. The carbohydrate last meal pattern produced a reduction in the amplitude of glucose excursions of 1.02 mmol/L in the study by Yong et al., 40.3 mg/dL in Shukla et al., and 2.16 mmol/L in Imai et al. All of these studies employed a randomized crossover design and included a carbohydrate first and a carbohydrate last arm. Yong et al. and Imai et al. measured glucose using a continuous glucose monitoring system in mmol/L while Shukla et al. used blood samples and assessed in whole blood using a quantitative enzyme photometry cassette in mg/dL [13, 16, 18]. Despite methodological differences, the direction of effect was consistent across all three studies, suggesting robustness across techniques.

Additionally, four studies examined the incremental area under the curve (iAUC) for postprandial glucose excursions, all studies (100%), consistently reporting significant reductions following the carbohydrate last meal pattern [13, 14, 16, 18]. Yong et al. reported a reduction in iAUC_0‐3h_ of 286 mmol/L following the carbohydrate last meal pattern for dinner. Kuwata et al. showed that the carbohydrate last meal pattern reduced iAUC_15‐240min_ by 0.83 mmol/L. Similarly, Imai et al. produced a 212 mmol/L reduction in iAUC_0‐3h_ and Shukla et al. produced a 3578 mmol/dL reduction following the carbohydrate last meal pattern. All of these studies used the same design (randomized controlled crossover) The duration of the studies varied: 3 days [13, 14, 16] and 5 days [18].

Incremental glucose peaks were assessed in three studies, each study (100%) showing a significant decrease with the carbohydrate‐last meal pattern [13, 14, 16]. Shukla et al. produced a reduction of 40.3 mmol/dL in incremental glucose peaks following the carbohydrate last meal pattern compared to the carbohydrate first meal pattern, and Imai et al. produced a reduction of 1.74 mmol/L for the same measure. These studies were similar in design, all randomized crossover trials with a carbohydrate first and a carbohydrate last arm over a 3‐day period.

### 3.6 Other Outcomes

A summary of other outcomes can be found in Table 5. Two studies investigated insulin iAUC and all studies (100%) reported significant reductions with the carbohydrate‐last approach [14, 16]. Shukla et al. found that iAUC_0–180 min_ for insulin was 24.8% lower following the carbohydrate last meal pattern compared with the carbohydrate first pattern. these studies were randomized crossover trials with a carbohydrate first and a carbohydrate last arm. All studies assessed insulin via serum samples, though assay methods differed., Shukla et al. use a quantitative immunoradiometric assay and Kuwata et al. used a Lumi pulse presto insulin assay [14, 15, 16] Despite this variability, results were consistent, suggesting the robustness of outcome measure across laboratory techniques.

**TABLE 5:** The effect of macronutrient ordering interventions on other reported outcomes and whether it was statistically significant in participants with type 2 diabetes or gestational diabetes mellitus.

Outcome	Imai et al. (2013) [13]	Kuwata et al. (2016) [14]	Murugesan et al. (2025) [15]	Shukla et al. (2017) [16]	Tricò et al. (2016) [17]	Yong et al. (2022) [18]
Insulin iAUC	NM	↓*	NM	↓**	NM	NM
C‐Peptide iAUC	NM	↓*	NM	NM	NM	NM
GLP‐1 iAUC	NM	↑*	NM	↑*	NM	NM
GIP iAUC	NM	↑*	NM	NM	NM	NM
Gastric emptying rate	NM	↓*	NM	NM	NM	NM

*Note:* Statistical significance, ***p < 0.001, **p < 0.01, *p < 0.05.

Abbreviations: NM, Not Measured; NS, not significant.

One study also found significant decreases in iAUC for C‐peptide, GIP, and gastric emptying rate following the carbohydrate last meal patter [14]. Furthermore, two studies measured GLP‐1 iAUC and similarly both studies (100%) reported a significant increase under the carbohydrate‐last condition [14, 16]. These two studies were similar in design; both were randomized crossover trials conducted over 3 days with a carbohydrate first and a carbohydrate last arm. Shukla et al. measured the plasma concentration of GLP‐1 using an electro chemiluminescent assay kit, while Kuwata et al. used a human total GLP‐1 assay kit.

## 4 Discussion

The aim of this review was to systematically examine the evidence to date regarding the effectiveness of macronutrient ordering as an intervention to lower postprandial glucose levels in individuals with diabetes. Overall, the results indicate that a carbohydrate last meal pattern is effective at lowering postprandial glucose levels. Additionally, there is evidence to suggest that a carbohydrate last meal pattern is effective at lowering insulin, C‐peptide, and GIP, and increasing GLP‐1 in individuals with diabetes.

These findings suggest that manipulating the sequence of macronutrient ingestion may represent a simple, safe and practical strategy to improve postprandial glycaemic control in individuals with diabetes. All of the studies reported that a carbohydrate‐last meal pattern significantly reduced postprandial glucose excursions. These consistent reductions in postprandial glucose can be explained by well‐established physiological mechanisms. The effect of macronutrients on glucose tolerance is dependent on their ability to delay the rate of gastric emptying [10]. Gastric emptying is the delivery of the gastric contents, including liquids, digestible solids, and indigestible food residues, into the duodenum [19]. Gastric emptying is important in modulating the rate at which glucose is delivered and absorbed in the small intestine. Fat has been shown to be the most potent macronutrient in slowing gastric emptying, and protein has a smaller but similar effect [10, 20, 21, 22]. Therefore, by consuming fat or protein before carbohydrates, the slowing of gastric emptying reduces the speed of glucose absorption, thereby attenuating the postprandial glucose and insulin peaks observed across studies [10]. Beyond delaying gastric emptying, changing the order of macronutrient consumption may exert glucose‐lowering effects by stimulating the release of gut hormones such as GLP‐1, GIP, cholecystokinin, and peptide YY which inhibit gastric emptying and appetite [10]. GLP‐1 and GIP are two incretin hormones that have a glucose‐dependent and dose‐dependent stimulatory effect on pancreatic β cells [10]. By stimulating insulin secretion from these pancreatic β cells, GLP‐1 and GIP lower PPG [23]. In addition, GLP‐1 suppresses glucagon secretion which further delays gastric emptying [9]. Cholecystokinin and peptide YY exert their glucose‐lowering effects by delaying gastric emptying and decreasing appetite [10]. In addition to these mechanisms, improvements in insulin sensitivity and reductions in postprandial insulin clearance may represent additional mechanisms contributing to the observed reductions in postprandial hyperglycaemia [24]. Previous studies have demonstrated that consuming protein or fat prior to carbohydrate ingestion can enhance β‐cell glucose sensitivity and modestly reduce postprandial insulin clearance, thereby improving the efficiency of insulin action and contributing to improved glucose tolerance [24]. These mechanisms provide a plausible physiological explanation for the consistent reductions in postprandial glucose excursions observed across the studies included in this review.

The findings from the included studies in adults with diabetes are consistent with research on macronutrient ordering in other populations. For example, in a 2018 study by Faber et al. [25], the effect of a carbohydrate last meal pattern on postprandial glucose levels in children with type 1 diabetes mellitus (T1DM) was examined. This study was an open label, within‐subject crossover study conducted in the Netherlands. Twenty patients aged between 7 and 17 years diagnosed with T1DM for more than 1 year were recruited. The study duration was 3 days. The study demonstrated that the consumption of protein or fat before carbohydrates resulted in lower postprandial glucose levels, consistent with the results from the included studies in this review. Similarly, the PATTERN study from 2020 [26], showed that eating vegetables before carbohydrates reduced postprandial glucose excursions in 16 healthy individuals (13 male and 3 female) aged between 18 and 24 of Chinese ethnic background. Furthermore, various studies have been conducted to investigate the effects of ‘preloading’ meals with proteins and/or fats. In a 2020 systematic review of meal sequences, seven studies looking at preloading fat or protein before carbohydrates in individuals with T2DM were identified. Of these seven studies, preloading suppressed PPG excursions in six studies, enhanced GLP‐1 secretion in all studies, and enhanced GIP secretion in five studies [9]. However, preloading meals with protein or fat is considered a less acceptable dietary approach than macronutrient ordering. This is because nutrient preloads increase the daily caloric intake, which may lead to an increase in body weight and this extra food might be expensive [10]. These convergent findings across populations and study design strengthen the validity of the results from this review and support the broader applicability of carbohydrate‐last meal sequencing.

The studies included in this review had several methodological strengths. Most studies employed randomization in their design, which minimized between‐subject variability and reduced bias, thereby strengthening the reliability of the observed effects. The study design and methods used were, for the majority, clearly defined. Most of the studies employed some method to measure the adherence to the test meals and most did frequent glucose measurements. All of the studies used realistic meals in terms of food and quality enhancing the real‐world applicability of the study and all measured baseline characteristics, including fasting plasma glucose. The studies also had several notable limitations. All of the studies used a small sample size (sample size ranged from 12 to 63 participants) which limits statistical power and generalizability and the majority of them were over a short duration (3–5 days), which restricts ability to evaluate longer term adherence to this dietary approach and the sustained impact on glycaemic control. The studies typically only assessed acute postprandial responses rather than long term glycaemic outcomes. There was also considerable variation in the tools used for measuring glucose levels (e.g., capillary vs. CGM) and the units that glucose levels were reported in (mmol/L vs. mg/dL) making comparison between studies more difficult. None of the studies assessed any other factors that could impact glucose levels such as physical activity and socio‐economic status. The majority (four of seven) of studies used a randomized crossover design. With a crossover design, the two arms cannot be treated as independent groups thereby making it difficult to examine differences between groups. The differences in the duration of the included studies (3 days to 43 weeks) also makes it difficult to make comparisons across each study. Finally, heterogeneity in populations and meal protocols complicates interpretation and limits the generalizability of the findings.

To the authors' knowledge, this is the first systematic review to focus specifically on the effects of macronutrient ordering on postprandial glucose levels in people with diabetes. Broad search terms with no exclusion criteria were used across multiple databases to capture as many relevant papers as possible. The omission of grey literature may increase the risk of publication bias. A robust systematic approach involving two independent reviewers was used to select the final papers. However, as highlighted by Nesti et al., gathering all available evidence in this area is challenging due to the different key words used by different research groups (e.g., macronutrient/food/meal order/sequence) [10].

Currently, the evidence base is limited by small sample sizes, short study durations, and heterogeneity in study design and populations; however, the findings highlight the potential of carbohydrate‐last meal sequencing as a low‐cost, low‐risk behavioural intervention. Future research is needed and should focus on longer term trials with larger sample sizes, longer durations, standardized meal protocols and standardized glucose measurements to determine longer term effect on glucose control. Further randomized controlled trials should be carried out to show independent effects.

## 5 Conclusions

This systematic review demonstrates that macronutrient is effective at lowering postprandial glucose levels in individuals with diabetes. The consistent improvements in postprandial glycaemia across diverse populations are supported by plausible physiological mechanisms, including delayed gastric emptying and gut hormone release. Overall, the results provide evidence for the effectiveness of this dietary strategy and show that changing the sequence of food intake by eating carbohydrates last represents a simple, practical and low‐cost strategy that could be readily adopted to improve postprandial glucose control in diabetes.

## Author Contributions

**Hannah O'Hara:** writing – review and editing, supervision. **Jayne Woodside:** validation, writing – review and editing, supervision. **Charlotte Sterling:** investigation, validation, writing – review and editing, supervision. **Rebecca McKenzie:** conceptualization, methodology, investigation, formal analysis, writing – original draft, writing – review and editing, visualization.

## Funding

This work is from a PhD studentship which was funded by the Department for the Economy in Northern Ireland.

## Ethics Statement

The authors have nothing to report.

## Conflicts of Interest

The authors declare no conflicts of interest.

## Supporting information


**Appendix S1:** Full Database search strategy
**Table S1:** GRADE Assessment

## Data Availability

Data sharing not applicable to this article as no datasets were generated or analysed during the current study.
