# Supplementary material for: The Impact of Macronutrient Ordering on Postprandial Glycaemic Control in Diabetes: A Systematic Review
Source: Endocrinol Diabetes Metab. 2026 Apr 27;9(3):e70228. doi: 10.1002/edm2.70228 (PMC13121093; doi:10.1002/edm2.70228)
Supplement: Supplementary file 1 — Appendix S1: Full Database search strategy Table S1: GRADE Assessment [file EDM2-9-e70228-s001.docx]

**Supplementary Materials**

**Supplementary Appendix 1: Full Database search strategy**

Database: MEDLINE, Web of Science, Embase

Date searched: February 2025

Limits Applied: None

1     macronutrient*.mp.

2     Food/

3     meal*.mp.

4     order*.mp.

5     sequenc*.mp.

6     postprandial glyc*.mp.

7     glyc* excursion*.mp.

8     postprandial hyperglyc*.mp.

9     Hyperglycemia/

10    exp Diabetes Mellitus/

11    1 OR 2 OR 3

12    4 OR 5

13    6 OR 7 OR 8 OR 9

14    10 AND 11 AND 12 AND 13

*mp. indicates a multi-purpose search field including title, abstract, subject heading word, and keyword heading word

**Supplementary Table 1: GRADE Assessment**

| **Outcome** | **No. of Studies** | **Study Design** | **Risk of Bias^1^** | **Inconsistency** | **Indirectness** | **Imprecision^3^** | **Publication bias^4^** | **Certainty of evidence** |
| --- | --- | --- | --- | --- | --- | --- | --- | --- |
| **Postprandial Glucose Excursions** | 6 | Randomised Controlled Trials (4 crossover, 2 parallel) | Low/ some concerns- downgrade -1 | Consistent | Mild indirectness^2^- downgrade -1 | Imprecise- downgrade -1 | Undetected | Low |
| **Insulin** | 2 | Randomised controlled trials (crossover) | Low/Some concerns- downgrade -1 | Consistent | Mild indirectness^2^- downgrade -1 | Imprecise- downgrade -1 | Undetected | Low |
| **C-Peptide** | 1 | Randomised controlled trial (crossover) | Some concerns- downgrade -1 | Not assessable (single study) | Not assessable (single study) | Imprecise- downgrade -1 | Undetected | Low |
| **GLP** | 2 | Randomised controlled trials (crossover) | Low/Some concerns- downgrade -1 | Consistent | Indirect- downgrade -1 | Imprecise- downgrade -1 | Undetected | Low |
| **GIP** | 1 | Randomised controlled trial (crossover) | Some concerns- downgrade -1 | Not assessable (single study) | Not assessable- (single study) | Imprecise- downgrade -1 | Undetected | Low |

1. Some studies had some methodological concerns which resulted in some concerns in the RoB2 assessment; therefore, certainty was downgraded one level.
2. Minor differences in study design (e.g. variations in meal composition and study populations) resulted in downgrading one level for indirectness.
3. All studies had a relatively small sample size resulting in downgrading one level for imprecision
4. The small number of included studies limited formal assessment of publication bias, although none was suspected.
